# Severe resistant hypocalcemia in multiple myeloma after zoledronic acid administration: a case report

**DOI:** 10.1186/1752-1947-8-353

**Published:** 2014-10-23

**Authors:** Adrian P Noriega Aldave, Shikha Jaiswal

**Affiliations:** 1 UAB Health Center Montgomery, 2055 East South Boulevard, Suite 202, Montgomery, AL 36116, USA; 2 East South Boulevard, Suite 202, Montgomery, AL 36116, USA

**Keywords:** Hypocalcemia, Multiple myeloma, Vitamin D deficiency, Zoledronic acid

## Abstract

**Introduction:**

Hypercalcemia is one of the most common metabolic abnormalities encountered in any form of malignancy. Hypocalcemia, however, is a rare manifestation, especially in cancers with bone involvement. Here we present a case of hypocalcemia in a patient with multiple myeloma that was refractory to treatment.

**Case presentation:**

A 73-year-old African American woman recently diagnosed with multiple myeloma, presented with a 2-day history of fever, vomiting and hypocalcemia. Ten days prior to admission she received zoledronic acid, Velcade^®^ (bortezomib), Revlimid^®^ (lenalidomide) and dexamethasone. Treatment was started with intravenous antibiotics and calcium gluconate boluses. After 24 hours of treatment her calcium level became undetectable (<5mg/dL). Continuous intravenous calcium gluconate infusions in addition to boluses were started. She remained persistently hypocalcemic and eventually developed tonic–clonic seizures. Vitamin D levels were found to be low and intravenous paricalcitol was initiated, which improved her calcium level.

**Conclusions:**

Underlying vitamin D deficiency can precipitate severe hypocalcemia in patients with multiple myeloma receiving bisphosphonates. This warrants baseline screening for vitamin D deficiency in these patients.

## Introduction

Multiple myeloma (MM) is a malignant proliferation of plasma cells, characterized by the presence of monoclonal immunoglobulin in serum and urine. Zoledronic acid (ZA) is a standard part of the treatment, as patients with MM usually have hypercalcemia secondary to bone destruction. However, some cases of hypocalcemia have been reported when bisphosphonates were used in patients with vitamin D deficiency [1-3]. Our case differs in the fact that our patient with MM developed severe hypocalcemia just after receiving her first dose of ZA.

## Case presentation

A 73-year-old African American woman with diabetes mellitus, hypertension, and recently diagnosed immunoglobulin G MM, presented with 2 days’ history of fever, nausea, vomiting and dizziness. She was found to be obtunded in our Emergency department and was intubated for airway protection. She was febrile (39.33°C, 102.8°F), but hemodynamically stable. She demonstrated mild respiratory distress, had rales and wheezes on auscultation, and edema in both lower extremities. Initial laboratory results (Table 1) revealed pancytopenia, elevated creatinine and corrected calcium of 7.3mg/dL, other electrolytes were within normal limits. A chest radiograph revealed cardiomegaly and pulmonary edema. Ten days prior to admission she received one dose of ZA, three doses of bortezomib, and seven doses of daily lenalidomide in addition to weekly dexamethasone. A clinical diagnosis of neutropenic fever, acute renal failure and severe hypocalcemia was made. Treatment was started with intravenous vancomycin and cefepime. She was also given calcium gluconate boluses for her hypocalcemia.

**Table 1:** Comparison of laboratory parameters at first chemotherapy dose, admission and hospital stay

**CHEMISTRY**	**Biochemistry 1 day before chemotherapy**	**1 day after zoledronic acid and Velcade**^**® **^**(bortezomib) administration**	**3 days after admission**	**1 day after paricalcitol initiated**
Corrected calcium (mg/dL)	10.3	7.3	<5 (undetectable)	8.4
Ionized calcium (mmol/L)	–	–	0.66	1.13
Creatinine (mg/dL)	0.99	1.69	8	4.8
Glomerular filtration rate (mL/minute/1.73m^2^)	>60	36	6	–
Vitamin D (ng/mL)	–	–	13	–

Laboratory results not done.

**Figure 1:**
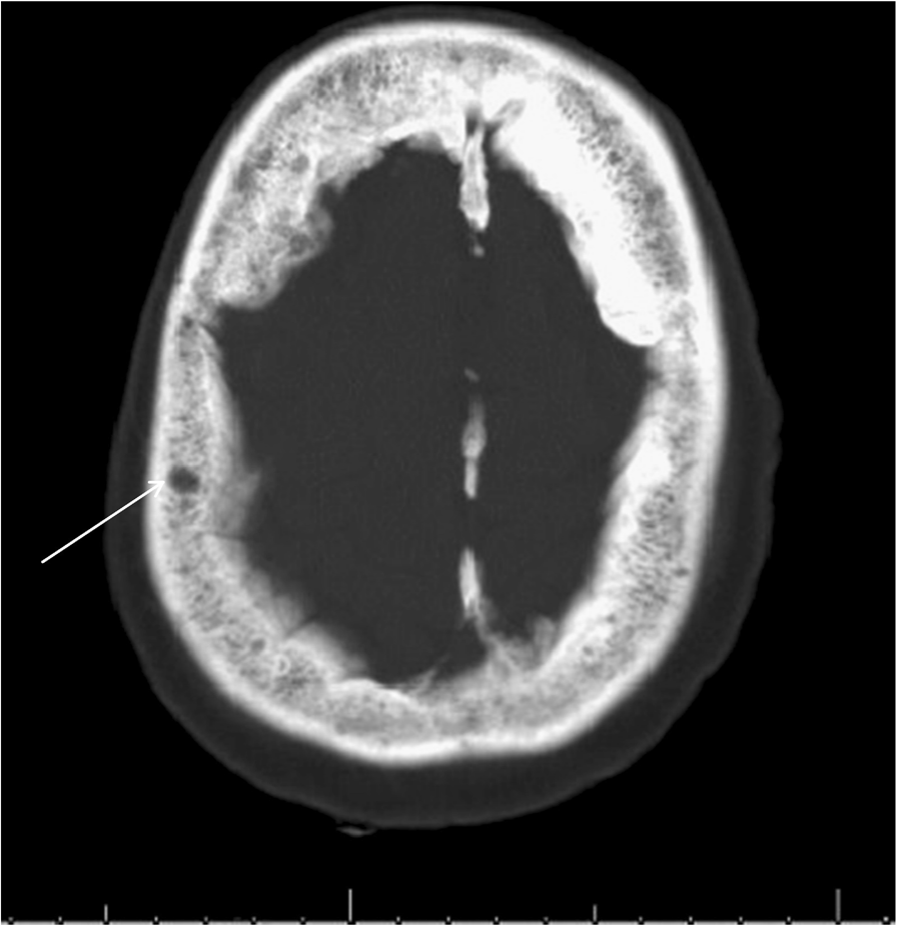
**Computed tomography of brain (bone window).** Computed tomography brain scan showing multiple lytic lesions in the calvarium (arrow).

Even after 24 hours of treatment, her mental status did not improve. She remained febrile, her kidney functions worsened, and her calcium level became undetectable (<5mg/dL) and ionized calcium was 0.66mmol/L (see Table 1). She was started on continuous intravenous calcium gluconate infusion in addition to boluses, which still did not increase her calcium levels. We then tested for vitamin D levels which were found to be low (13ng/mL). The value of vitamin D was measured on the day in which renal function was most impaired (creatinine 8mg/dL). Intravenous paricalcitol was initiated, which did improve her calcium level, but, it did not revert to normal. Despite aggressive calcium replacement she remained persistently hypocalcemic and eventually developed tonic–clonic seizures. An electrocardiogram revealed a QT interval of 500ms using Bazett’s formula. Her neuroimaging, however, was normal except for several small calvarium lytic lesions (Figure 1). Her family eventually made the decision to focus on comfort measures only, following which, she had a cardiac arrest and died on hospital day 17.

## Discussion

ZA is a long-acting bisphosphonate used for supportive therapy in MM and bone metastasis. It has a safety profile similar to that of pamidronate, but because of ease of administration, it is preferred over the latter. In the long run, both pamidronate and ZA have been shown to reduce skeletal related events, and decrease need for irradiation [4].

Bisphosphonates are generally well tolerated, although they are occasionally associated with adverse events namely hypocalcemia, nephrotoxicity, pancytopenia and osteonecrosis [5-7].

Renal dysfunction is an undesired side effect that can occur after intravenous infusion of bisphosphonate [5]. In this case, her kidney functions were normal before starting chemotherapy, but a week after initiating ZA, she developed acute renal failure. This may also have been exacerbated by the MM nephropathy, but arguably her kidney functions had remained stable from the time of the diagnosis until she got her first dose of ZA.

All bisphosphonates can cause hypocalcemia, regardless of their method of administration, although this is infrequently found to be a clinically symptomatic problem [7,8]. Of note, symptomatic hypocalcemia after ZA is rarely reported.

Hypovitaminosis D and concurrent dexamethasone and ZA administration have been identified as independent risk factors for severe hypocalcemia when metastatic tumors are treated with ZA [9]. Corticosteroids decrease blood calcium levels by suppression of intestinal calcium absorption, depression of vitamin D activity and reabsorption of calcium in renal tubules.

There are some case reports about patients with MM with unrecognized vitamin D deficiency, who developed hypocalcemia following ZA that improved only after vitamin D replacement [1]; as can also be appreciated in this case.

## Conclusions

Some studies have reported up to 150% increase in mortality, among critical care unit patients, with ionized calcium levels below 0.8mmol/L [10]. Our patient had ionized calcium of 0.66mmol/L. Unfortunately, the level of vitamin D is not part of routine screening in patients receiving bisphosphonates for cancers. This case therefore serves to remind us that careful clinical and biochemical evaluation is required before administration of bisphosphonates in cancers to avoid undesired side effects; and vitamin D levels should be a part of screening before administering more potent and longer acting bisphosphonates, such as ZA.

## Consent

Written informed consent was obtained from the patient for publication of this case report and any accompanying images. A copy of the written consent is available for review by the Editor-in-Chief of this journal.

## Abbreviations

MM: Multiple myeloma; ZA: Zoledronic acid.

## Competing interests

The authors declare that they have no competing interests.

## Authors’ contributions

APNA wrote the introduction, case description, table and corrected discussion of the manuscript, SJ wrote discussion and conclusions. Both authors read and approved the final manuscript.
